# Stability of the Helical TomoTherapy Hi·Art II detector for treatment beam irradiations

**DOI:** 10.1120/jacmp.v15i6.4897

**Published:** 2014-11-08

**Authors:** Karin Schombourg, François Bochud, Raphaël Moeckli

**Affiliations:** ^1^ Institute of Radiation Physics University Hospital Centre and University of Lausanne Grand‐Pré 1 CH‐1007 Lausanne Switzerland

**Keywords:** tomotherapy detector, stability, treatment beam

## Abstract

The Hi·Art II Helical TomoTherapy (HT) unit is equipped with a built‐in onboard MVCT detector used for patient imaging and beam monitoring. Our aim was to study the detector stability for treatment beam measurements. We studied the MVCT detector response with the 6 MV photon beam over time, throughout short‐term (during an irradiation) and long‐term (two times 50 days) periods. Our results show a coefficient of variation ≤1% for detector chambers inside the beam (excluding beam gradients) for short‐ and long‐term response of the MVCT detector. Larger variations were observed in beam gradients and an influence of the X‐ray target where degradation was found. The results assume that an ‘air scan’ procedure is performed daily to recalibrate the detector with the imaging beam. On short term, the detector response stability is comparable to other devices. Long‐term measurements during two 50‐day periods show a good reproducibility.

PACS numbers: 87.55.ne, 87.55.Qr

## I. INTRODUCTION

The Helical TomoTherapy (HT) Hi·Art II unit (Accuray Inc., Sunnyvale, CA) is equipped with an onboard single‐row detector allowing megavolt (MV) computed tomography (CT) imaging of the patient.^(1)^ Patient position may thus be verified just before the dose delivery by matching the planning kilovolt (kV) CT with the MVCT.^(2)^ In addition, the MVCT detector also measures fluence transmitted through the patient during the treatment. There is interest in using these data for transit dosimetry (dose delivery verification). Transit dosimetry is based on measurements performed during patient irradiation. Different methods were proposed for dose delivery verification using transit measurements, namely comparison against a dose precalculated at the patient entrance or patient exit (transit dose), or a 2D or 3D dose reconstruction.^(3)^ The dosimetry may be performed with point dose detectors or 2D tools. For example, 2D measurements may be carried out by electronic portal imager devices (EPIDs).^(4)^ Some 3D dose reconstructions methods were proposed, using the transit dose.^5^, ^6^, ^7^, ^8^, ^9^, ^10^ These techniques depend on the MVCT detector's short‐ and long‐term stability. Beside transit dosimetry, there are various reasons for which one is interested in the stability of the MVCT detector response. The MVCT detector can be used in routine for beam monitoring and treatment unit alignment verifications.^11^, ^12^ For example, the TomoTherapy Quality Assurance (TQA) tools^13^, ^14^ allow us to analyze the treatment beam profile with the MVCT detector. This profile is then compared to a reference profile previously acquired. For such application, the stability of the MVCT detector response has to be known.

A procedure called ‘air scan’ (explained later) is performed every day in the HT unit before treatments start. This procedure should ensure the MVCT detector's stability for image‐guided radiotherapy (IGRT). Some authors have simulated the MVCT detector^(15)^ and others studied the MVCT images.^16^, ^17^, ^18^, ^19^ The aim of our study was to verify that the MVCT detector is stable with the 6 MV treatment beam, for transit dosimetry. We performed short and longer term irradiations of the MVCT detector, in order to evaluate its temporal response with static and rotating beams.

## II. MATERIALS AND METHODS

Different types of MVCT detectors are available on the HT units. Our device is provided with 640 xenon‐filled CT channels housed in an aluminum box (General Electric, Fairfield, CT). In the following text we will use the word detector as a synonym with ‘MVCT detector’. The detector's surface‐to‐isocenter distance is 56.95 cm and the surface‐to‐photon source distance is 141.95 cm. The radius of curvature of the detector does not correspond to the circle on which the linac rotates. This is known as the out‐of‐focus of the detector.^(15)^ The detector CT channels are separated by tungsten septal plates, which are not in the line of divergence of the beam. Therefore the collimation properties vary as a function of the lateral distance from the beam axis. The amount of scatter produced increases towards the edges of the detector, thus the detector response is larger in the edges than in the center.^(15)^ Consequently, there is a signal dip at the center of the measured beam profiles. The beam is measured by 540 CT channels; the other CT channels do not receive any signal from the beam because the beam width is smaller than the detector's lateral width. The spatial resolution at the isocenter along the x‐axis (transverse plane) is 0.74 mm.

Two sealed monitor ion chambers are located in the machine head upstream of the Y jaws.^(12)^ They are used to monitor the beam output.^(12)^ Monitor 1 (mon1) measures radiation at the center of the beam. The radius of the collection surface is about 7 cm. As recommended by Accuray Inc., a procedure called “air scan” is performed daily before beginning of the treatments. This procedure collects the detector signal after irradiation with the MV imaging beam and compares it against reference values. The gain of each CT channel is corrected accordingly in order to fit the reference values.

We studied the short‐ and long‐term stability of this detector by irradiating it with static and rotating 6 MV beams. At each irradiation, the multileaf collimator (MLC) leaves were closed during the first 10 s to allow for the dose rate to stabilize, as in usual treatments.^(12)^ The linac pulse rate is 300 Hz. Both the data acquisition frequency of mon1 and the detector were 30 Hz. The TQA tools were used to extract the detector and mon1 data. All CT channels signals were normalized by mon1 to take into account the effects of dose‐rate variations^(20)^ for each acquired pulse. For each irradiation procedure, a detector mean beam profile was calculated as the average signal over the irradiation duration. All the field widths given in the text are defined at the isocenter. Analysis was performed for all CT channels inside of the beam (the beam penumbra was excluded and defined here at 60% of the maximum signal measured). For practical reasons, in the rest of this paper we show graphics with results for three CT channels (number 350, 385, and 550) of the detector. They were arbitrarily chosen at representative positions of the beam profile (near the center, in the shoulder, and in the edge of the beam profile), in high‐ and low‐ signal gradient regions, but they are fully representative of the behavior of any of the other CT channels.

### A. Experiments performed

All irradiations were performed with a $40\times 1\;{\text{cm}}^{2}$ beam, except for irradiations with rotating gantry where a $40\times 5\;{\text{cm}}^{2}$ beam was also used. Measurements were performed on the tomotherapy SN137 (tomo1) of our department before the installation of a dose control system (dose servo) (DCS), except when explicitly stated.

#### A.1 Short‐term stability— irradiations with static gantry

The detector short‐term stability was evaluated by five repeated irradiations during the same day, with the gantry at 0° (static irradiation). The beam‐on time was 10 min. In addition, the response during a static irradiation of 120 s was evaluated over a 100‐day period, where the irradiations were repeated 17 times.

#### A.2 Short‐term stability — irradiations with rotating gantry

Further 10 min irradiations were performed with the gantry rotating (24 s rotation period). These measurements were repeated three times during the same day.

#### A.3 Irradiations with the DCS

Five min irradiations with rotating gantry were performed after the installation of the DCS on tomo1, two‐and‐a‐half years after the beginning of this study. An identical irradiation was performed on tomotherapy SN290 (tomo2) of the department, also equipped with a DCS.

#### A.4 Long‐term stability

Long‐term stability of the detector was determined by performing 17 measurements with static beams over a 103‐day period. The irradiation time was 130 s (10 s to stabilize the beam and 120 s with all MLC leaves open). We used static beams to exclude variations due to gantry rotation, already studied in the short‐term stability tests. During that period of time, the target was replaced.

### B. Parameters studied

The parameters defined hereafter were calculated for all the CT channel signals normalized by mon1, on an acquired pulse‐by‐pulse basis. In the following text, we will use the term ‘normalized signal’ as equivalent to ‘signal normalized by mon1’. The coefficient of variation (CV) of a CT channel i signal was defined by Eq. (1):

(1) $$CV_{i}=100\cdot \frac{sd_{i}}{{\bar{x}}_{i}}$$

with ${\text{sd}}_{i}$ being the standard deviation (SD) of the signal acquired at time t, and $x_{i}$ the average value over the irradiation duration. The *CV* does not have any units, which allows using it for comparisons within data from different devices. The difference between the maximum and the minimum signals during one irradiation was obtained by Eq. (2):

(2) $$\text{max}\_{\text{min}}_{i}=100\cdot \frac{{\text{max}}_{i}-{\text{min}}_{i}}{{\bar{x}}_{i}}$$

where ${\text{max}}_{i}$ and ${\text{min}}_{i}$ are the maximum and minimum of the normalized signal over the entire irradiation for the chamber i. The deviation from the average signal value (DA) was calculated for each CT channel i by Eq. (3):

(3) $$DA_{i}=100\cdot \frac{x_{i}-{\bar{x}}_{i}}{{\bar{x}}_{i}}$$

where $x_{i}$ is the value of the normalized signal acquired at time t. For each CT channel, we defined the superior and inferior maximal deviations from the average values, ${\text{SAD}}_{i}$ and ${\text{IAD}}_{i}$, by Eqs. (4), (5):

(4) $${\text{SAD}}_{i}=100\cdot \frac{{\text{max}}_{i}-{\bar{x}}_{i}}{{\bar{x}}_{i}}$$

(5) $${\text{IAD}}_{i}=100\cdot \frac{{\text{min}}_{i}-{\bar{x}}_{i}}{{\bar{x}}_{i}}$$

Similarly to *CV, DA* does not have any units, but may be either negative or positive and so its minimal and maximal values give an idea about the symmetry of the signal variations with respect to the mean value. *DA(t)* represents angular variation more explicitly than CV. The CV is more representative of all signal variations occurring during the studied period. To calculate the long term CV for each CT channel, we used the same definition as above (i.e., the SD of the mean normalized CT channel values acquired at different days divided by the normalized signals average over the long‐term period).

## III. RESULTS

### A. Short‐term stability — irradiations with static gantry

**Figure 1:**
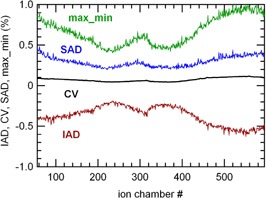
Short‐term stability (10 min, $40\times 1\;{\text{cm}}^{2}$ static irradiation): mean values of $\text{max}\_{\text{min}}_{i}$, ${\text{SAD}}_{i},{\text{CV}}_{i}$, and ${\text{IAD}}_{i}$ of the normalized signals as a function of the CT channels.

Normalized signals on the whole detector were relatively stable with time. The maximal ${\text{SAD}}_{i}$ and ${\text{IAD}}_{i}$ over all studied CT channels were +0.6% and −0.6%, respectively (Fig. 1). The $\text{max}\_{\text{min}}_{i}$ values were between 0.5% and 1.1%. The ${\text{DA}}_{i}$ values were within ±0.6%. The ${\text{CV}}_{i}$ were below 0.12% (static $40\times 1\;{\text{cm}}^{2}$ beam). The values were not symmetrical with respect to the central detector chambers. The four other static measurements performed the same day gave similar results.

### B. Short term stability — irradiations with rotating gantry

#### B.1 Irradiations with the 1 cm beam

**Figure 2:**
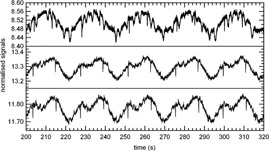
Zoom of three detector CT channel signals (350, 385, and 550 from bottom to top) normalized by mon1, as a function of the gantry angle.

**Figure 3:**
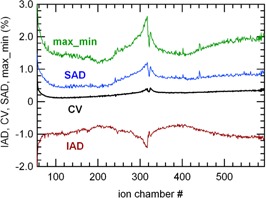
$\text{Shax}\_{\text{min}}_{i}$, ${\text{SAD}}_{i},{\text{CV}}_{i}$, and ${\text{IAD}}_{i}$ as a function of the CT channels, for the same irradiation as in Fig. 2.

A typical tomotherapy beam profile presents a signal dip at the center, which is specific to the detector.^(15)^ During the rotational irradiation, mon1 counts were within ±1.5% of the average value (DA), due to angular variation. This angular variation is generally up to 2% on treatments units not yet equipped with dose servo.^(12)^ The detector chambers signals still present an angular variation after normalization by mon1 (Fig. 2); the ${\text{DA}}_{i}$ were within ±1%. The $\text{max}\_{\text{min}}_{i}$ values varied from 1.2% to 2.8%, the larger values being on the central chambers (Fig. 3). The ${\text{CV}}_{i}$ were below +0.5%. The maximal ${\text{SAD}}_{i}$ and ${\text{IAD}}_{i}$ were +1.2% and −1.4%, respectively. Other measurements performed during the same day gave similar results. In a second analysis of the same irradiation, the signals of all CT channels were integrated and the resulting integrated signal was normalized by mon1. The resulting angular variation decreased with respect to those observed in the individual normalized chambers signals (Fig. 2) and remained within 0.5%.

#### B.2 Irradiations with the 5 cm beam

In the $40\times 5\;{\text{cm}}^{2}$ rotating beam irradiation, mon1 had a DA of about 0.8%. The normalized CT channels signals showed an angular variation with rotation similar to the results obtained with the 1 cm beam. However the ${\text{DA}}_{i}$ values were lower — from ±0.4% to ±0.6%. The ${\text{CV}}_{i}$ were within 1%. The larger values were at the detector center and for chambers 480 to 595. The maximal ${\text{SAD}}_{i}$ and ${\text{IAD}}_{i}$ were +1.3% and −1.8%, respectively. The $\text{max}\_{\text{min}}_{i}$ values were between 0.7% and 2.8%. A summary of the short‐term responses is presented in Table 1.

**Table 1:** Short‐term stability (10 min irradiations): summary of the detector response for static and rotating beams.

	*CVi (%)*	*DAi (%)*	*SADi (%)*	*IADi (%)*
$40\times 1\;{\text{cm}}^{2}$ static gantry (10 min)	0.04 to 0.12	±0.6	0.2 to 0.6	−0.6 to −0.2
$40\times 1\;{\text{cm}}^{2}$ rotating gantry (10 min)	to 0.70	±1.0	0.4 to 1.5	−2.0 to −1.7
$40\times 5\;{\text{cm}}^{2}$ rotating gantry (10 min)	0.10 to 0.70	±0.6	0.3 to1.3	−1.8 to −0.3

#### B.3 Irradiations with the DCS

**Figure 4:**
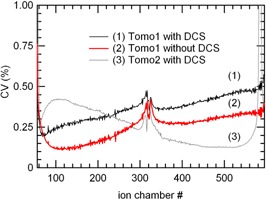
Irradiations with the DCS and short‐term stability (5 min, $40\times 1\;{\text{cm}}^{2}$ rotating beam): the ${\text{CV}}_{i}$ of two different detectors (tomo1 and tomo2) are presented. For comparison, the ${\text{CV}}_{i}$ for a similar irradiation performed on tomo1 without the DCS at a different time is also shown.

When the DCS was used, the CV were well within 1%, similarly to results without the DCS, except for channels 56, 57, 58, and 582–587 (most lateral channels) on tomo2 which were between 1% and 2% (Fig. 4). The CV of tomo2 detector varied with chamber position but with an inverted tendency respectively to tomo1 detector.

### C. Long‐term stability

**Figure 5:**
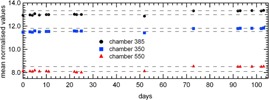
Long‐term stability over 103‐day time period ($40\times 1\;{\text{cm}}^{2}$ static gantry): normalized mean values of three detector CT channels as a function of time. A linac/X‐ray target replacement occurred between Day 52 and Day 72.

**Figure 6:**
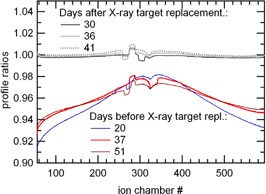
Six detector profiles acquired at different moments in the life time of the X‐ray target, divided by the profile acquired 11 days after X‐ray target replacement (all profiles were normalized by the respective mon1). The beam profiles were modifying in the edges as the X‐ray target was wearing. The overall mean detector values were larger after the replacement (> 2%).

Figure 5 shows mean normalized signals of three CT channels as a function of time over the 103‐day period. An X‐ray target replacement was completed on the treatment unit between Day 52 and Day 72. After the X‐ray target substitution, an increase in the mean values of the CT channels signals normalized by mon1 was registered. The mean beam profile measured on the detector had also a different shape. Figure 6 shows beam profile ratios acquired at different days before and after the X‐ray target replacement. The ratios were obtained by dividing each beam profile by the profile measured 11 days after X‐ray target replacement. The shape modification is similar to findings by other authors.^(21)^ The long‐term ${\text{CV}}_{i}$ calculated respectively during the first and the second period of time were well within 1%. The long‐term ${\text{CV}}_{i}$ calculated over the entire 103‐day period were within 3%. Besides, the short‐term ${\text{CV}}_{i}$ (i.e., over the 2 min irradiations) during the first period were larger with respect to the second period; they varied from 0.2% to 0.7% during the first period and from 0.1% to 0.3% during the second period, respectively.

## IV. DISCUSSION

We verified the temporal stability of our Hi·Art II xenon‐filled detector with the 6 MV treatment beam over a short and a long term time period. Because the dose rate of a tomotherapy unit without dose servo varies with the gantry angle and, furthermore, a dose drift may occur, we normalized the detector data by mon1 signal obtained with the same acquisition frequency. This rendered the data independent of dose‐rate variations in a first approximation. Several authors studied stability of other imaging devices used for transit dosimetry as, for instance, camera‐based EPIDs. Their short‐term variation in response was reported as less than 1% (1 SD).^(3)^ This is similar to the CV obtained for our detectors for chambers inside of the beam (excluding measurements in beam penumbra and, in one case, in the most lateral channels of the detector, where the variation was between 1% and 2%). In tomotherapy treatments, many small beams are used to deliver the dose to the target. For dose reconstruction purposes, the dosimetric impact of the variable CV should be investigated on an entire clinical treatment. Regarding rotational procedures, normalization of the detector signal by mon1 did not remove all angular variation. For the 1 cm beam, the mon1‐normalized signals still show an angular variation, which depends of the chamber position. By normalizing the integrated signal from all the detectors to mon1, beam profile variations were accounted for and apparent angular variation was reduced. This effect could be partly explained by the difference in the collection volumes of mon1 and the CT channels (mon1 collection volume is significantly larger and, due to the geometry, the detector chambers do not measure the signal at the same position of the beam). Currently, tomotherapy units are being equipped with a dose servo control system designed to stabilize the dose rate against angular variation. Experiments performed on both treatment units after being equipped with the DCS showed that the detector signals still contain an angular variation, however smaller, when mon1 did not vary with gantry angle. We did not find an explanation for this.

Our long term measurements were performed during a time period divided in two by an X‐ray target replacement. It was shown by other authors,^(21)^ who used a different measurement device, that the beam profile is changing a few percent in the beam edges as the X‐ray target is close to its end‐of‐life. We analyzed measurements of the periods before and after the X‐ray target replacement separately. The long‐term CV were well within 1% respectively before and after the target change (the transition is not included in this variation). Literature reports a 1% to 2% (1 SD) response variation for camera‐based EPIDs during a one‐year period.^(3)^ Other types of EPID, like the amorphous‐silicon (a‐Si) or flat‐panel, have a response variation of 0.5% (1 SD) over a two‐year period^(3)^ with 6 MV photon beams. In addition, X‐ray target aging seems to affect the short‐term response during irradiation of the CT channels. The CVs were larger the few weeks before the X‐ray target replacement.

The detector is monitored daily by the ‘air scan’ procedure using the MV imaging beam. This should ensure its stability for IGRT purposes. Our measurements were all performed within this context and, thus, are linked to the daily use of the ‘air scan’ procedure. A future study may also analyze the detector response with very attenuated small beam segments. It must also be mentioned that there are other types of detectors available on the tomotherapy units, for which temporal response may also be studied.

## V. CONCLUSIONS

We studied the stability of a HT Hi·Art II xenon‐filled MVCT detector with the 6 MV treatment beam. Short‐term measurements showed that the detector response inside the fan beam has stability comparable to other imaging detectors (excluding the beam penumbra). Long‐term measurements showed good reproducibility during two 50‐day periods.

## ACKNOWLEDGMENTS

The authors would like to thank Dirk Verellen, Universitair Ziekenhuis Brussel, for his comments on the manuscript. The authors would also like to thank Bob Cravens, Accuray Inc, for his technical support with the TQA tools, and Tanja Wolf for providing us with the TQA tools for our research purposes.
